# Soluble IL‐2R: A potential therapeutic target for mitochondrial dysfunction in post‐COVID fatigue syndrome

**DOI:** 10.1002/ctm2.70507

**Published:** 2025-10-13

**Authors:** Laura P. Brown, Jai Joshi, Kate Kosmac, Douglas E. Long, Logan Scott, Salim El‐Amouri, Ashley A. Montgomery‐Yates, Anna G. Kalema, Jamie L. Sturgill, Hemendra Vekaria, Patrick G. Sullivan, Dylan Wilburn, Panagiotis Koutakis, Christine M. Latham, Christopher S. Fry, Philip A. Kern, Benjamin F. Miller, Esther E. Dupont‐Versteegden, Ahmed Ismaeel, Kirby P. Mayer, Yuan Wen

**Affiliations:** ^1^ Department of Physiology University of Kentucky Lexington Kentucky USA; ^2^ Center for Muscle Biology University of Kentucky Lexington Kentucky USA; ^3^ Department of Physical Therapy Augusta University Augusta Georgia USA; ^4^ Division of Pulmonary Critical Care and Sleep Medicine Department of Internal Medicine University of Kentucky Lexington Kentucky USA; ^5^ Department of Microbiology Immunology, and Molecular Genetics University of Kentucky Lexington Kentucky USA; ^6^ Spinal Cord and Brain Injury Research Center University of Kentucky Lexington Kentucky USA; ^7^ Department of Neurosurgery Medical University of South Carolina Charleston South Carolina USA; ^8^ Department of Cell Biology University of Texas Southwestern Medical Center Dallas Texas USA; ^9^ Department of Biology Baylor University Waco Texas USA; ^10^ Department of Public Health University of West Florida Pensacola Florida USA; ^11^ Department of Athletic Training and Clinical Nutrition University of Kentucky Lexington Kentucky USA; ^12^ Division of Endocrinology Department of Internal Medicine University of Kentucky Lexington Kentucky USA; ^13^ Aging and Metabolism Research Program Oklahoma Medical Research Foundation Oklahoma City Oklahoma USA; ^14^ Oklahoma City VA Medical Center Oklahoma City Oklahoma USA; ^15^ Department of Physical Therapy University of Kentucky Lexington Kentucky USA; ^16^ Division of Biomedical Informatics Department of Internal Medicine University of Kentucky Lexington Kentucky USA

Dear Editor,

We demonstrate that elevated soluble IL‐2 receptor (sIL2R) may directly cause mitochondrial dysfunction in skeletal muscle, providing novel mechanistic insight into post‐COVID fatigue. This finding reveals sIL2R as a potential mediator linking systemic immune dysregulation to muscle pathology and suggests a potential therapeutic target for this debilitating condition.

Post‐COVID fatigue affects millions worldwide, yet its underlying mechanisms remain poorly understood. While previous studies documented mitochondrial abnormalities in post‐acute sequelae of COVID (PASC) patients,^1^, ^2^ the specific circulating factors responsible for muscle dysfunction remain speculative. We hypothesised that T‐cell‐derived inflammatory mediators^2^, ^3^, ^4^ directly impair muscle mitochondrial function, causing the persistent fatigue characteristic of PASC.

We recruited 11 PASC patients (characteristics summarised in Table S1) experiencing fatigue >4 weeks post‐infection and 17 healthy community‐dwelling adults as controls (no documented history of COVID and no reported hospitalisation within 12 months). PASC participants required positive SARS‐CoV‐2 test, mild‐to‐moderate illness without hospitalisation or supplemental oxygen, and persistent fatigue ≥4 weeks post‐infection. Participants with recent hospitalisations or medications affecting muscle function were excluded. There were no statistically significant differences between the healthy (*n* = 17, 47.1% female, 42.2 ± 16.4 years, body mass index [BMI] 27.6 ± 3.3 kg/m^2^) and PASC (*n* = 11, 50.0% female, 34 ± 10.2 years, BMI 27.7 ± 4.7 kg/m^2^) groups in terms of BMI (*p* = .96) or sex distribution (*p* = .22). An overview of the study participant sample size distribution across all analyses is shown in Figure 1. PASC participants demonstrated a statistically significant (*p* < .05) 16.4% lower average distance on standardized 6‐min walk tests^5^ despite normal muscle mass and strength (Figure 2A‒F). This functional impairment occurred without detectable muscle atrophy, suggesting metabolic rather than structural deficits.

**FIGURE 1:**
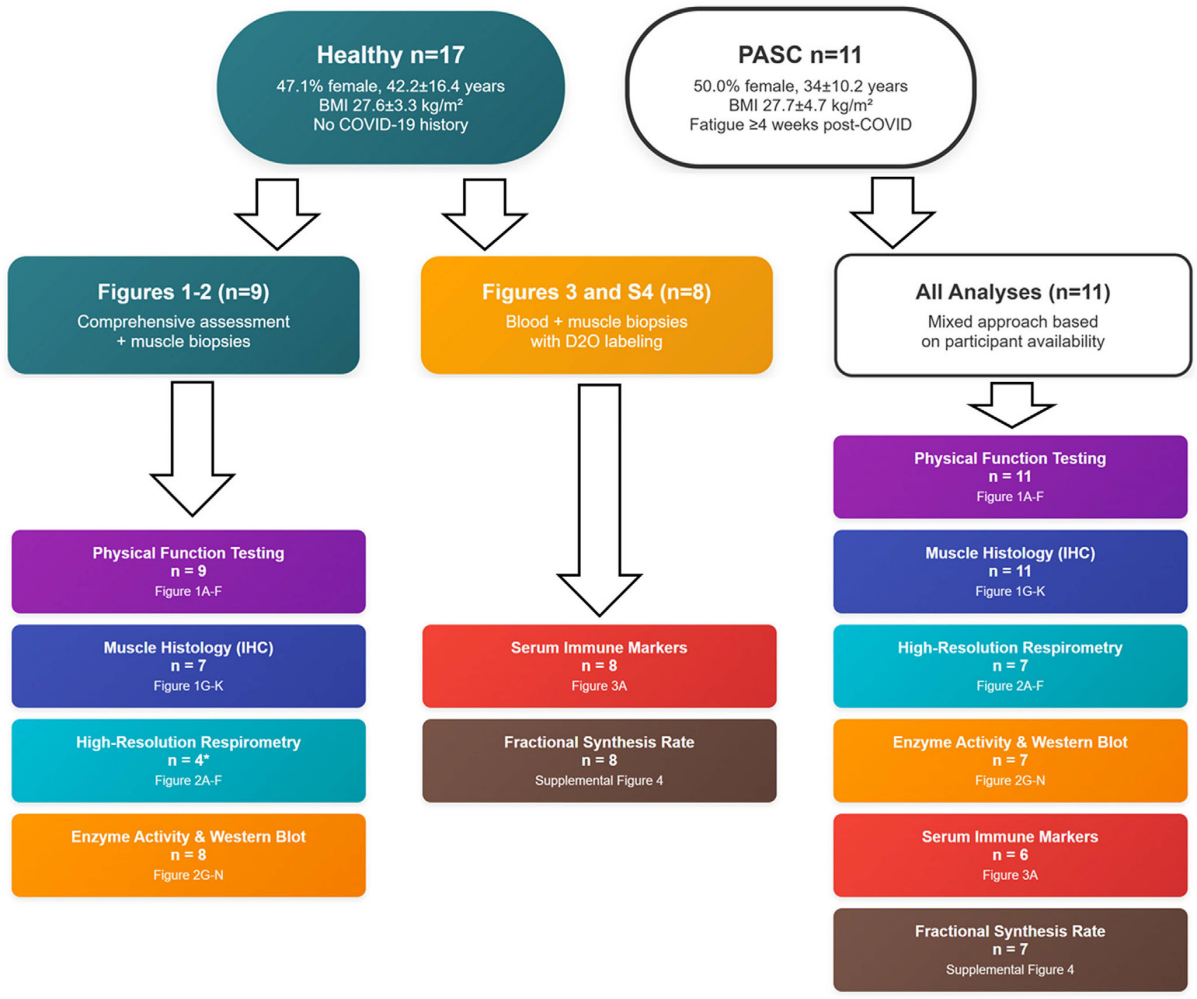
Participant allocation and sample size distribution across analyses. Flowchart showing the allocation of healthy controls (*n* = 17) and post‐acute sequelae of COVID (PASC) patients (*n* = 11) across different experimental analyses. Healthy participants followed two distinct protocols: comprehensive assessment with muscle biopsies for mitochondrial function and histological analyses (*n* = 9), or blood samples and muscle biopsies after D2O consumption without physical assessments (*n* = 8). PASC participants used a mixed approach with varying sample sizes based on consent and availability. Colour coding indicates analysis type: purple (physical function), blue (histology/IHC), teal (respirometry), orange (enzyme/Western blot), red (immune markers) and brown (synthesis rate). ^*^High‐resolution respirometry was limited to *n* = 4 healthy controls due to technical requirements (immediate access to respirometry equipment, tissue preservation solution and mitochondrial respiration medium) and was validated against previously published control data using identical methodology. Due to sample quantity limitations, not all experiments could be conducted on all subjects. Statistical comparisons between healthy and PASC groups showed no significant differences in demographic measures: age (*p* = .18), body mass index (BMI; *p* = .96) and sex distribution (*p* = .22). Abbreviations: IHC, Immunohistochemistry; OXPHOS, Oxidative Phosphorylation; PBS, Phosphate Buffered Saline.

**FIGURE 2:**
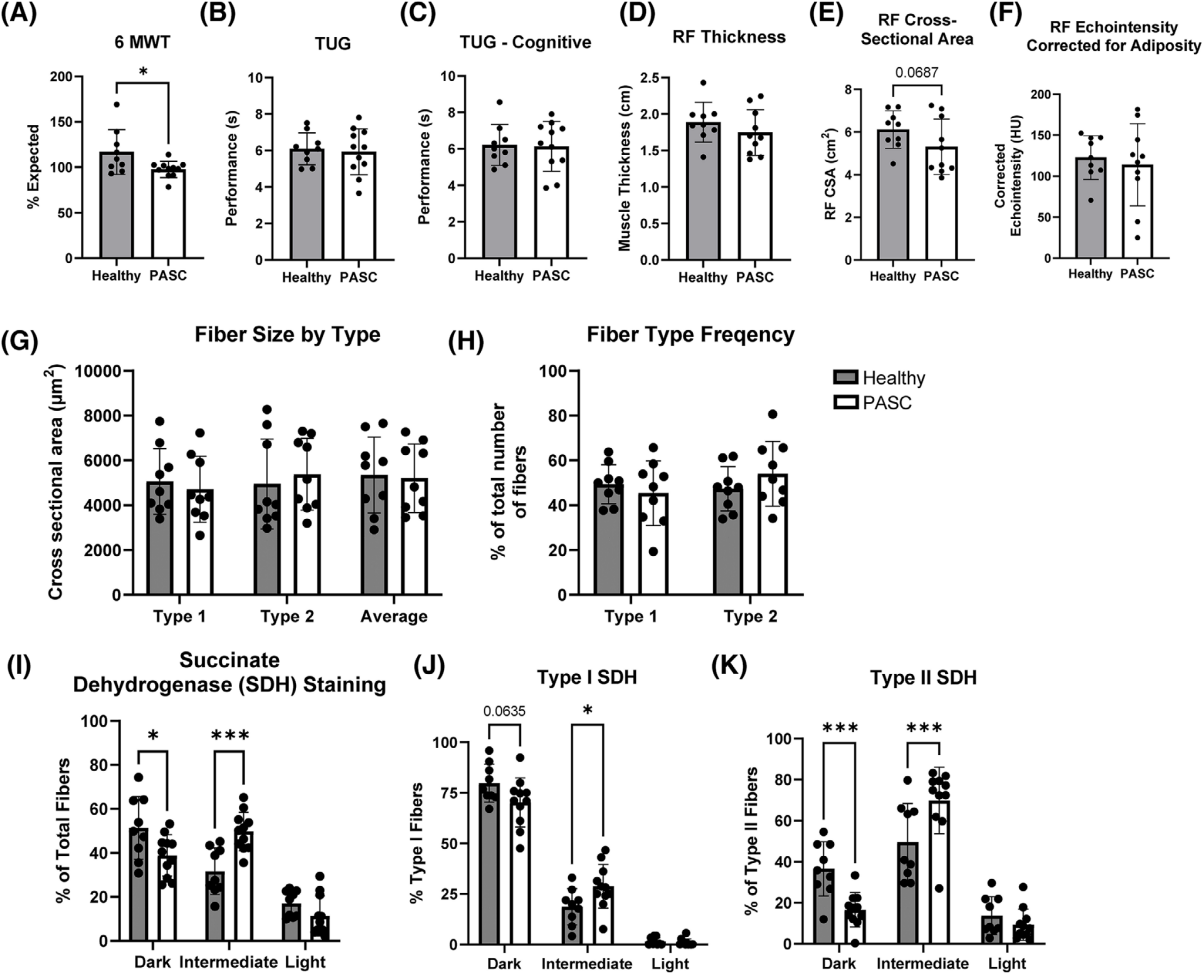
Muscle function testing, ultrasonography and histological indicators of altered muscle fibre energetics within vastus lateralis biopsies from participants with post‐ acute sequelae of COVID (PASC) compared to healthy individuals. (A) Percent of expected 6‐ min walk testing (6 MWT) was significantly reduced in PASC subjects compared to healthy controls. (B and C) Timed up and go (TUG) tests both with and without simultaneous cognitive testing in healthy and PASC showing no significant differences. (D‒F) Both rectus femoris (RF) thickness and echointensity did not differ significantly between healthy and PASC, although RF cross‐sectional area showed a trend towards difference. (G‒K) Quantification of muscle fibre size, fibre type and mitochondrial activity/succinate dehydrogenase (SDH) within biopsies from PASC (*n* = 11) participants compared to healthy adults (*n* = 9). Data expressed as mean ± standard deviation (SD). (G) Fibre type specific cross‐sectional area (CSA) from PASC participants compared to healthy, showing no significant differences. (H) Frequency of fibres expressing type I or type II MyHC within muscle from PASC versus healthy adults, with no significant differences. (I) Quantification of overall muscle fibre SDH histochemistry in muscle showing a trending increase in dark fibres (*p* = .0606) and significantly higher number of intermediate fibres in PASC (*p* < .01). (J and K) Quantification of SDH histochemistry within individual fibre types showing significant differences in staining intensity distribution. *N* = 9 healthy and 11 PASC. ^*^ *p* < .05, ^**^ *p* < .01, ^***^ *p* < .001, and trending is defined as .05 < *p* ≤ .1.

Muscle biopsies suggested mitochondrial dysfunction in PASC patients as indicated by histological markers of muscle energetics (Figure 2G‒K). High‐resolution respirometry (HRR) demonstrated ∼30% reduction in mitochondrial oxygen consumption predominantly driven by complex I impairment (Figure 3A‒F). For high‐resolution respirometry specifically, only four of our 17 healthy subjects could be scheduled for biopsies with immediate access to respirometry equipment, tissue preservation solution and mitochondrial respiration. To address this limitation and validate our HRR findings, we compared the healthy control data against our previously published control data,^6^ using identical methodology, confirming no significant differences (Figure S1). Biochemical analyses confirmed significantly reduced citrate synthase and cytochrome C oxidase activities, while electron microscopy revealed mitochondrial structural abnormalities including membrane ruptures and increased autophagosome formation (Figure S2). Western blot analysis showed marked decreases in all mitochondrial respiratory complex proteins (Figure 3J‒N). Critically, mitochondrial protein synthesis rates remained normal (Figure S3A), indicating that reduced mitochondrial content may have resulted from increased degradation rather than impaired biogenesis.

**FIGURE 3:**
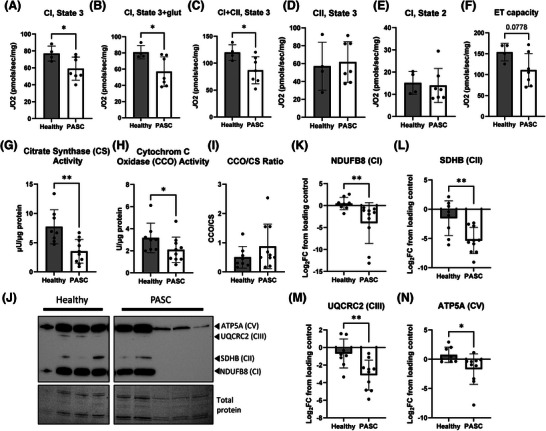
Mitochondrial respiration, enzyme activity and OXPHOS protein abundance between healthy and post‐acute sequelae of COVID (PASC) participants. (A‒D) Respiration is significantly lower for complex I (CI) state 3 (B), state3 + glutamate (C) and complex I + II (CI + CII) (D), but unchanged in complex I state 2 (A) and complex II state 3 (F). (E) Electron transport (ET) capacity trending towards significance. (G) Citrate synthase (CS) activity is significantly reduced in PASC participants when compared to healthy. (H) Cytochrome C oxidase (CCO) activity is significantly reduced in PASC. (I) The ratio of CCO to CS is not significantly different. (J‒N) Western blot analysis and quantification of protein subunits in mitochondrial complexes showing significant reductions in PASC for complex I (NDUFB8, K), complex II (SDHB, L), complex III (UQRCR2, M) and complex V (ATP5A, N). Data expressed as mean ± standard deviation (SD) with symbols representing individual data points. ^*^ *p* < .05, ^**^ *p* < .01, and trending is defined as .05 > *p* ≤ .1.

To identify circulating factors responsible for this mitochondrial pathology, we analysed 65 immune mediators in patient serum. Soluble IL‐2 receptor (sIL2R) emerged as the most dramatically elevated factor, showing up to 5 log‐fold higher concentrations in PASC patients compared to controls (Figure 4A). This finding is particularly significant given sIL2R's established role as a marker of T‐cell activation and its association with severe COVID‐19 outcomes.^2^, ^3^, ^4^

**FIGURE 4:**
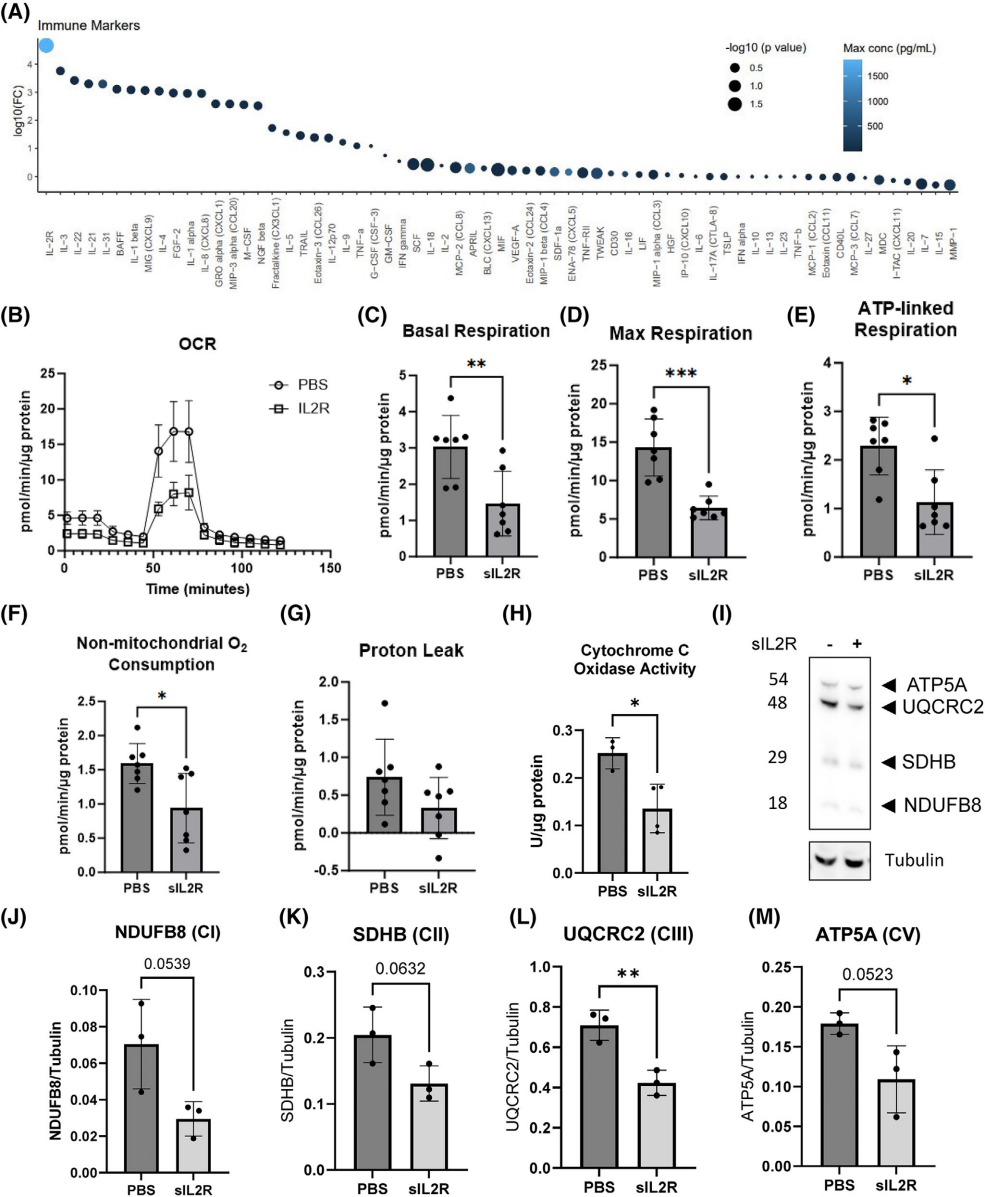
Soluble interleukin 2 receptor (sIL2R) is the highest inflammatory marker in post‐acute sequelae of COVID (PASC) and impairs myocyte mitochondrial function in vitro. (A) Dot plot showing log10 fold change of inflammatory markers in PASC patients when compared to healthy controls. sIL2R is the most significantly elevated systemic factor in PASC (largest dot, highest on vertical axis). Dot size negatively scales with *p* value (larger dots indicate higher statistical significance), and colour indicates the marker's highest concentration in serum (pg/mL), with lighter blue representing higher concentrations. *N* = 8 healthy and 6 PASC. (B) Oxygen consumption rate (OCR) is reduced in myotubes after sIL2R administration compared to PBS control (*n* = 7). (C‒F) Basal respiration, maximal respiration, ATP‐linked respiration and non‐mitochondrial oxygen consumption were all significantly lower in sIL2R‐treated cells compared to PBS controls. (G) Proton leak was not significantly different between sIL2R and control. (H) Cytochrome C oxidase activity was significantly reduced in sIL2R‐treated cells compared to PBS controls. (I) Representative Western blot analysis of C2C12 myotube lysates showing mitochondrial complex proteins with and without sIL2R treatment. (J‒M) Quantification of protein subunits showing trending decrease in complex I (NDUFB8, J), trending decrease in complex II (SDHB, K), significant decrease in complex III (UQRCR2, L) and trending decrease in complex V (ATP5A, M). Data expressed as mean ± standard deviation (SD). ^**^ *p* < .01, ^*^ *p* < .05, and trending is defined as .05 < *p* ≤ .1.

We directly tested sIL2R's effects on muscle mitochondrial function using C2C12 myotube cultures. Treatment with physiologically relevant sIL2R concentrations for 24 h caused significant reductions in basal, maximal and ATP‐linked respiration (Figure 4B‒H). Importantly, IL‐2 alone had no effect, demonstrating that sIL2R's actions are distinct from classical IL‐2 signalling pathways (Figure S3B,C). Mechanistic studies revealed that sIL2R treatment reduced mitochondrial complex III protein levels (Figure 4I‒M), consistent with our patient biopsy findings.

These results establish sIL2R as a potentially novel pathogenic factor in post‐COVID fatigue. The mechanism likely involves sIL2R binding to muscle cell surface receptors, such as β1‐integrins,^7^ or employing trans‐signalling pathways similar to other soluble cytokine receptors.^8^ This systemic‐to‐local pathway may explain how persistent immune activation following COVID‐19 infection translates into organ‐specific mitochondrial dysfunction.

Our findings suggest potential therapeutic implications. Unlike broad immunosuppression, targeting sIL2R offers a specific intervention that could preserve beneficial immune responses while addressing pathogenic inflammation. Potential approaches include neutralising antibodies, receptor antagonists or existing therapies such as intravenous immunoglobulin^9^ that may reduce sIL2R levels. Additionally, our work suggests that sIL2R levels may serve as a biomarker for PASC severity and treatment response.

The broader significance extends beyond COVID‐19. Post‐viral fatigue syndromes have been recognized for decades,^10^ including cognitive symptoms such as ‘brain fog^3^’, yet their mechanisms remained elusive. Our identification of the sIL2R‐mitochondrial dysfunction axis may explain similar conditions following other viral infections and could revolutionize treatment approaches for chronic fatigue syndromes.

The biggest limitation in this study was the small sample size, which limits statistical power, generalizability and precluded robust correlation analyses in our current study; however, the effect sizes were large and consistent across multiple independent measures. Additionally, reduced physical activity levels in PASC participants could potentially influence results, although several findings suggest this is not simple deconditioning: preserved muscle mass and strength, specific complex I‐predominant dysfunction pattern, direct sIL2R effects in vitro, and mitochondrial protein synthesis rates not different from healthy controls. The C2C12 cell model does not fully replicate human myocyte immunophenotype, and future studies with primary human myotubes would strengthen our findings. The exact molecular mechanisms of sIL2R's mitochondrial effects require further investigation, as do potential tissue‐specific differences in sensitivity.

## 1 CONCLUSION

We have identified elevated sIL2R as a possible cause of mitochondrial dysfunction in post‐COVID fatigue, providing novel mechanistic insight at the molecular level for this condition. Building upon previous studies documenting mitochondrial abnormalities in PASC, our preliminary findings suggest that sIL2R may be a specific circulating factor mediating muscle dysfunction. While these results require validation in larger studies, they may advance our understanding of PASC pathophysiology and suggest a potential, druggable target for therapeutic intervention. Given the prevalence of those affected by post‐COVID fatigue worldwide, these findings represent a critical step toward evidence‐based treatments for this debilitating condition.

## AUTHOR CONTRIBUTIONS

Laura P. Brown led manuscript preparation, data analysis and experiments. Douglas E. Long collected blood and performed functional tests. Philip A. Kern, Ashley A. Montgomery‐Yates and Anna G. Kalema collected muscle biopsies. Jai Joshi, Kate Kosmac, Hemendra Vekaria, Dylan Wilburn, Logan Scott, Salim El‐Amouri, and Christine M. Latham conducted experiments and analyzed data. Jamie L. Sturgill, Patrick Sullivan, Philip A. Kern, Panagiotis Koutakis, Christopher S. Fry and Esther E. Dupont‐Versteegden provided mentorship and scientific oversight. Ahmed Ismaeel performed experiments, analysed results and assisted with manuscript preparation. Kirby P. Mayer conceived the project and supervised overall direction. Yuan Wen designed experiments, guided research direction and contributed to manuscript writing. All the authors provided critical feedback and approved the final manuscript.

## CONFLICT OF INTEREST STATEMENT

Y.W. is the owner of MyoAnalytics, LLC. The other authors have no conflict of interests to report.

## ETHICS STATEMENT

This prospective, cross‐sectional study was approved by the University of Kentucky institutional review board. Participants were 18 years or older and provided informed consent, and the study adhered to the Helsinki Declaration.

## Supporting information



Supporting information

Supporting information

Supporting information

Supporting information

## Data Availability

Data will be made available upon reasonable request.
