# Supplementary material for: Soluble IL‐2R: A potential therapeutic target for mitochondrial dysfunction in post‐COVID fatigue syndrome
Source: Clin Transl Med. 2025 Oct 13;15(10):e70507. doi: 10.1002/ctm2.70507 (PMC12516084; doi:10.1002/ctm2.70507)
Supplement: Supplementary file 1 — Supporting information [file CTM2-15-e70507-s001.pdf]

**PASC participant characteristics**	**Results**	
Days since positive SARS-CoV-2 test	59.36±24.44 days	
Activity level prior to infection	High	2(18.18%)
	Intermediate	4(36.36%)
	Low	5(45.45%)
Change in activity	Reduced	8 (72.73%)
	No change	3 (27.27%)
Chronic myalgia since infection	Yes	3 (27.27%)

Inclusion criteria for PASC included subjective report of fatigue at time of enrollment and biopsy testing.

Supplemental table 1. PASC participant self reported characteristics.
Days since positive test is reported as average days and standard deviation. Activity level prior to infection, change in activity, and chronic myalgia since infection is reported as quantity reporting and percentage of all PASC participants who answered (n=11).
