# Supplementary material for: Soluble IL‐2R: A potential therapeutic target for mitochondrial dysfunction in post‐COVID fatigue syndrome
Source: Clin Transl Med. 2025 Oct 13;15(10):e70507. doi: 10.1002/ctm2.70507 (PMC12516084; doi:10.1002/ctm2.70507)
Supplement: Supplementary file 2 — Supporting information [file CTM2-15-e70507-s003.pdf]

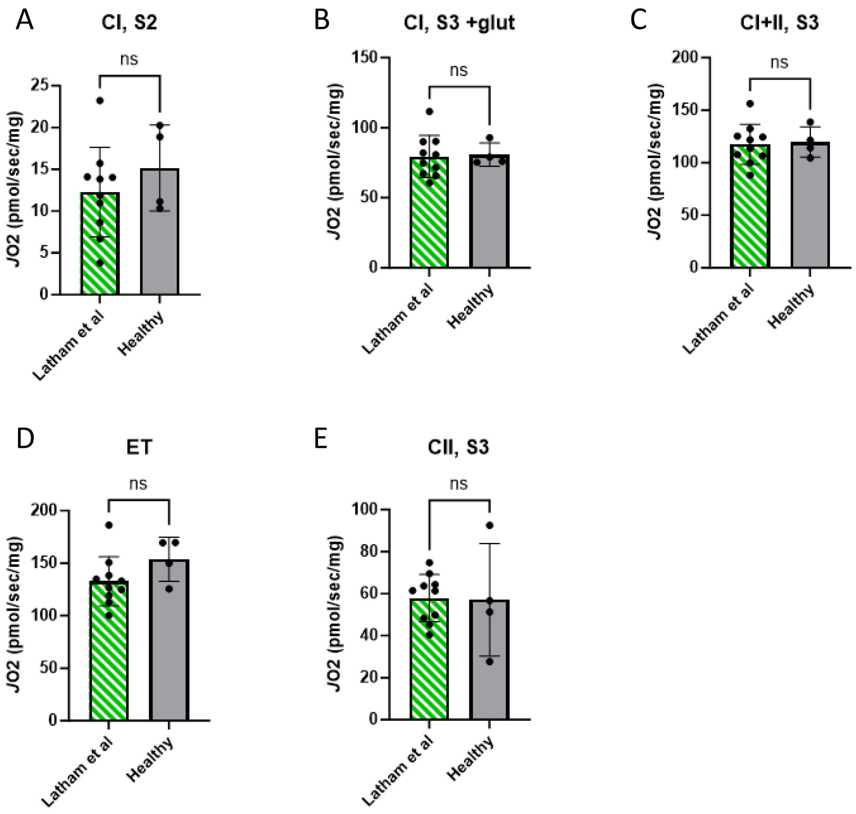

Supplemental Figure 1. Comparison of high resolution respirometry of our data (Healthy) vs previously published (Latham et al) data. There was no significant difference in complex I state 2 (**A**) complex I state 3 + glutamate (**B**), complex I+II state 3 (**C**), electron transport capacity (**D**), and complex II state 3 (**E**) respiration. For comparison, only Latham et al participants who were 18 years old or older with biopsy from non-injured limb were included. (Student T test)
