# Supplementary material for: Soluble IL‐2R: A potential therapeutic target for mitochondrial dysfunction in post‐COVID fatigue syndrome
Source: Clin Transl Med. 2025 Oct 13;15(10):e70507. doi: 10.1002/ctm2.70507 (PMC12516084; doi:10.1002/ctm2.70507)
Supplement: Supplementary file 3 — Supporting information [file CTM2-15-e70507-s004.pdf]

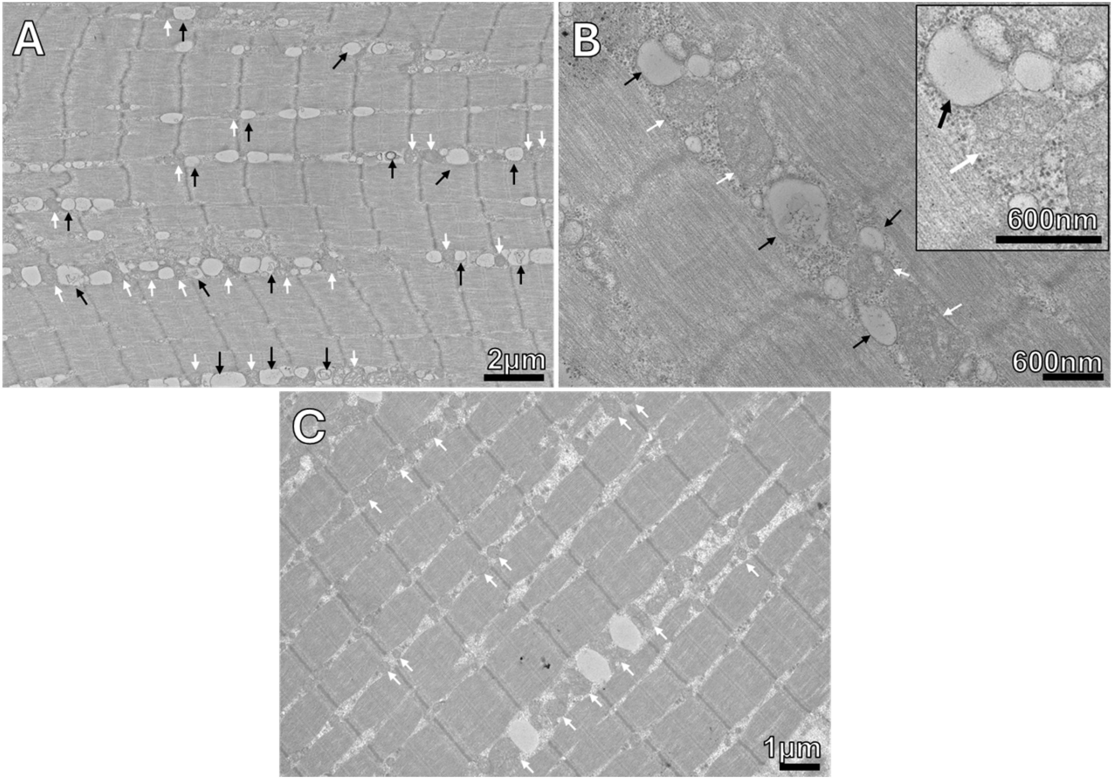

Supplemental Figure 2. Increased autophagosomes localized near mitochondria. Pannels **A** and **B** show representative micrographs of a PASC participant's skeletal muscle sarcomere and mitochondrial features at 2μm and 600nm. Compared to control muscle (**C**) PASC displayed fewer mitochondrial pairs at Z-discs and increased autophagosome (black arrows) localization near mitochondria (white arrows).
