# Supplementary material for: Soluble IL‐2R: A potential therapeutic target for mitochondrial dysfunction in post‐COVID fatigue syndrome
Source: Clin Transl Med. 2025 Oct 13;15(10):e70507. doi: 10.1002/ctm2.70507 (PMC12516084; doi:10.1002/ctm2.70507)
Supplement: Supplementary file 4 — Supporting information [file CTM2-15-e70507-s002.pdf]

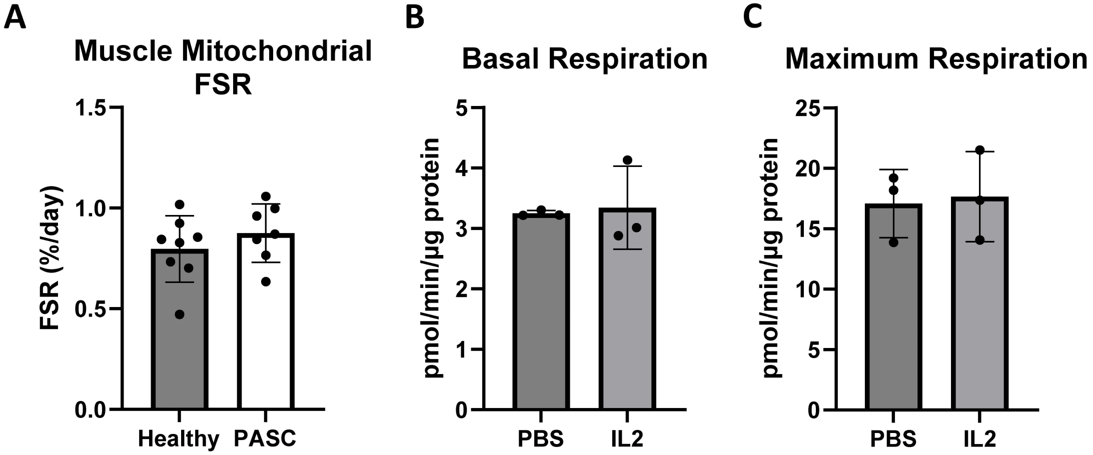

Supplemental Figure 3. Muscle mitochondria fractional protein synthesis (FSR) and cellular respiration after administration of IL2 on C2C12 myotubes. A. No differences in FSR (%/day) n = 8 healthy, 7 PASC. p = 0.2523 (Mann-Whitney U test). Basal respiration (**A**) and maximal respiration (**B**) is unchanged after IL2 is administered on C2C12 myotubes (N=3).
